# A Scoping Review of Gender Differences in Suicide in India

**DOI:** 10.3389/fpsyt.2022.884657

**Published:** 2022-05-20

**Authors:** Parvathy Ramesh, Peter J. Taylor, Rebecca McPhillips, Rajesh Raman, Catherine Robinson

**Affiliations:** ^1^School of Health Sciences, The University of Manchester, Manchester, United Kingdom; ^2^Department of Psychiatry, JSS Medical College, JSS University, Mysore, India

**Keywords:** suicide, gender differences, India, scoping review, mental health

## Abstract

**Introduction:**

Much of the published literature on suicide comes from high income countries. In countries such as India, female suicide rates exceed the global suicide rate and suicide rates found in their male counterparts. Results from previous studies indicate that factors related to suicide among men and women in India are different from those seen in high-income countries. To date, no reviews have considered the relationship between gender and suicide in India. Therefore, the aim of this scoping review is to provide a comprehensive understanding of existing literature reporting gender differences in suicide rates, methods, risk factors and antecedent factors in India by reviewing published studies.

**Method:**

A scoping review was conducted to map the existing literature on gender differences in suicide in India. To identify peer-reviewed publications, online databases PsycINFO and Embase were searched. The search terms were [suicid^*^ AND India^*^]. The searches took place in November 2020 and May 2021, with no language restrictions. Articles published from 2014 onwards from India were included. Reference lists of selected studies were searched for studies that could meet the inclusion criteria.

**Results:**

This review identified 17 studies that met the inclusion criteria. The ratio between women and men who die by suicide in India is much lower than in high-income countries. Hanging was found to be a more commonly used method of suicide among both men and women, in comparison to high-income countries where hanging is more common among men. This review also identified several gaps in the literature. There were few studies that examined suicide among transgender Indians. There was limited literature on gender differences in risk and protective factors for suicide. Limitations such as the omission of a lack of gender-based analyses in several studies and under-reporting of suicide rates were identified.

**Conclusion:**

Understanding suicide within the context of individual countries is essential in designing culture-appropriate suicide prevention strategies. This review identified an urgent need to establish and evaluate suicide surveillance systems in India. Furthermore, additional research is warranted to understand suicide among individuals who identify outside the gender binary, and gender-specific risk and protective factors.

## Introduction

Suicide is a major challenge to global public health. The World Health Organisation (WHO) estimates that around 800,000 suicide deaths occur worldwide every year, with an annual global age-standardised suicide rate of 10.5 per 100,000 population (1). Many countries lack suicide registration systems (2), which affects the accuracy of the estimated suicide rate and gender differences. Registration systems of good quality are more likely to be found in high-income countries (HICs); 95% of suicides in HICs are estimated through good-quality registration data from 39 countries, while only 8% of suicide data in low and lower middle-income countries (LMICs) are estimated through good quality registration data from 21 countries (2).

For those countries where suicide data is available, the suicide rate in men exceed those found in women (2). However, the suicide rate among men and women can differ by region and age group. Age-disaggregated data shows that in some parts of the world, female suicide rates are higher than the global average, and exceed those rates found in their male counterparts (1). Evidence gathered on antecedent factors, risk factors, and protective factors for suicide from HICs may not be applicable in LMICs, as the latter often have fewer healthcare resources and significantly different sociocultural factors (3, 4) that affect sex differences in suicide. Available data indicates that while males in HICs have a higher suicide rate (19.9 per 100,000) compared to males in LMICs (13.7 per 100,000), a reversed trend is observed in females. Rates of suicide are higher among females from LMICs compared to females in HICs (8.7 per 100,000 as compared to 5.7 per 100,000) (5). In LMICs, suicide rates in females comprise 43% of all suicides, while in HICs, this is 23% of all suicides (6). The male:female suicide ratio is lower for low-income regions in the South-East Asia Region (SEAR), with a male:female ratio of 1.57: 1. Due to under-reporting of data, it is possible that these rates could be higher, especially in South Asian countries (7).

India has a population of more than 1.3 billion, which is roughly one sixth of the world's population (8). India accounts for the majority (82%) of suicides among countries in the SEAR and has the highest suicide rate among all countries in the SEAR, thus making this an important country within the region for suicide prevention. In 2016, the suicide rate in India was 16.5/100,000, which was higher than the global average of 10.5/100,000 (1). The suicide rate in India for the 15–29 age group is 36.1 per 100,000 for females and 34.9 per 100,000 for males (2). Suicide was the leading cause of death in the age group of 15–39 years for women in India (9), thus emphasising the need to examine the epidemiology of suicide and its relation to gender in this country.

Scoping reviews are indicated when the aim is to provide an overview of existing literature and identify gaps in this literature, while a systematic review is conducted to appraise the strength of evidence for theories or treatments, confirm current practises in a field, identify conflicting results, and to understand the quality of existing studies (10). A limited amount of reviews to date have included a focus on gender and suicide in India. A systematic review on suicide in India had been conducted by Rane and Nadkarni (11). This review provided a general overview of suicide rates, methods and demographic factors of suicide decedents in India. Similarly, Jordans et al. (7) conducted a scoping review on suicide in South Asia (7). While this scoping review identified gender differences in suicide rates, gender differences in other factors such as methods, risk factors, antecedent factors were not assessed. Since there have been no previous reviews that specifically examined gender differences in suicide in India, a scoping review on gender differences in suicide in India would be an appropriate tool to examine the scope of literature and provide a contemporary overview of the studies available on this subject. The aim of this scoping review is to provide a comprehensive synthesis and understanding of the literature on gender differences in rates, methods, risk factors and antecedent factors for suicide in India.

## Method

### Definition

For the purpose of this review, suicide is defined as the act of deliberately killing oneself; gender is used to describe the characteristics of women and men that are socially constructed, while sex refers to those characters that are biologically determined (12). In this review, the term sex or gender has been used depending on the term the author of the cited paper has chosen for their study.

### Search Strategy

The Joanna Briggs Institute (JBI) guidelines (13) for scoping reviews were followed for this review. A scoping review of peer-reviewed publications was conducted to map the existing literature reporting gender differences in rates, methods, risk factors and antecedent factors for suicide in India. The studies in this review were expected to have diverse approaches to measurement of suicide data. In such cases, a meta-analysis is not recommended since genuine differences in effects may be obscured (14).

Published studies were identified using the following strategy. To identify peer-reviewed publications, online databases (PsycINFO and EMBASE) were searched, encompassing literature in the field of psychology, medicine, and health sciences. The search terms used were: [suicid^*^ AND India^*^]. The searches took place in November 2020 and May 2021, for studies that were published starting from May 1, 2014. Identification of relevant studies was based on screening the title and abstract of identified studies. Reference lists of identified studies were searched for studies that met inclusion criteria. The Preferred Reporting Items for Systematic Reviews and Meta-Analyses Extension Fillable Checklist (PRISMA-ScR) (15) was followed.

Including grey literature in a review can provide a more comprehensive view of the available evidence. While peer-reviewed literature ordinarily utilises a specific format, grey literature can differ in format and length, which makes their inclusion and analysis time- and resource-intensive (16). As the time required to review these documents and identify specific information within them would be considerable, it was beyond the scope of this review to include grey literature.

### Inclusion and Exclusion Criteria

Articles published from May 2014 onwards focussing on suicide in India were included. This date was selected to provide a recent overview of gender differences in suicide in India, since the most recent review examining suicide data in India by Rane and Nadkarni (11) covered studies before 2014. Studies were included if they examined suicide rates, suicide methods, risk factors for suicide, or antecedent factors for suicide in Indians and were conducted in India. Both quantitative and qualitative studies were eligible for inclusion. Papers were excluded if they did not provide relevant information on suicide in India or were published before 2014. Editorials, commentaries, and studies with data from newspapers were excluded.

During the initial screening of publications, articles were included if it was unclear whether the data examined suicide attempts or suicide deaths. The full text of these articles was then reviewed to determine whether the study examined self-harm, suicide attempts, or suicide deaths. Only data on suicide deaths from these studies were included for the narrative synthesis. Similarly, for those studies that only assessed gender differences for one variable of interest (for example, only rates of suicide were reported), the study was included, but only relevant information was extracted. PR screened studies and extracted data from selected studies. Any questions around study eligibility were jointly discussed and resolved by PR, PT, RM and CR. PR summarised the findings from the studies included.

### Data Extraction

Data from the studies were entered into a pre-piloted extraction form. The form included details on authors, year of publication, location of the study, study aims, period of data collection, method and type of data collected, suicide rates, methods, risk factors, and antecedent factors.

## Results

**Figure 1:**
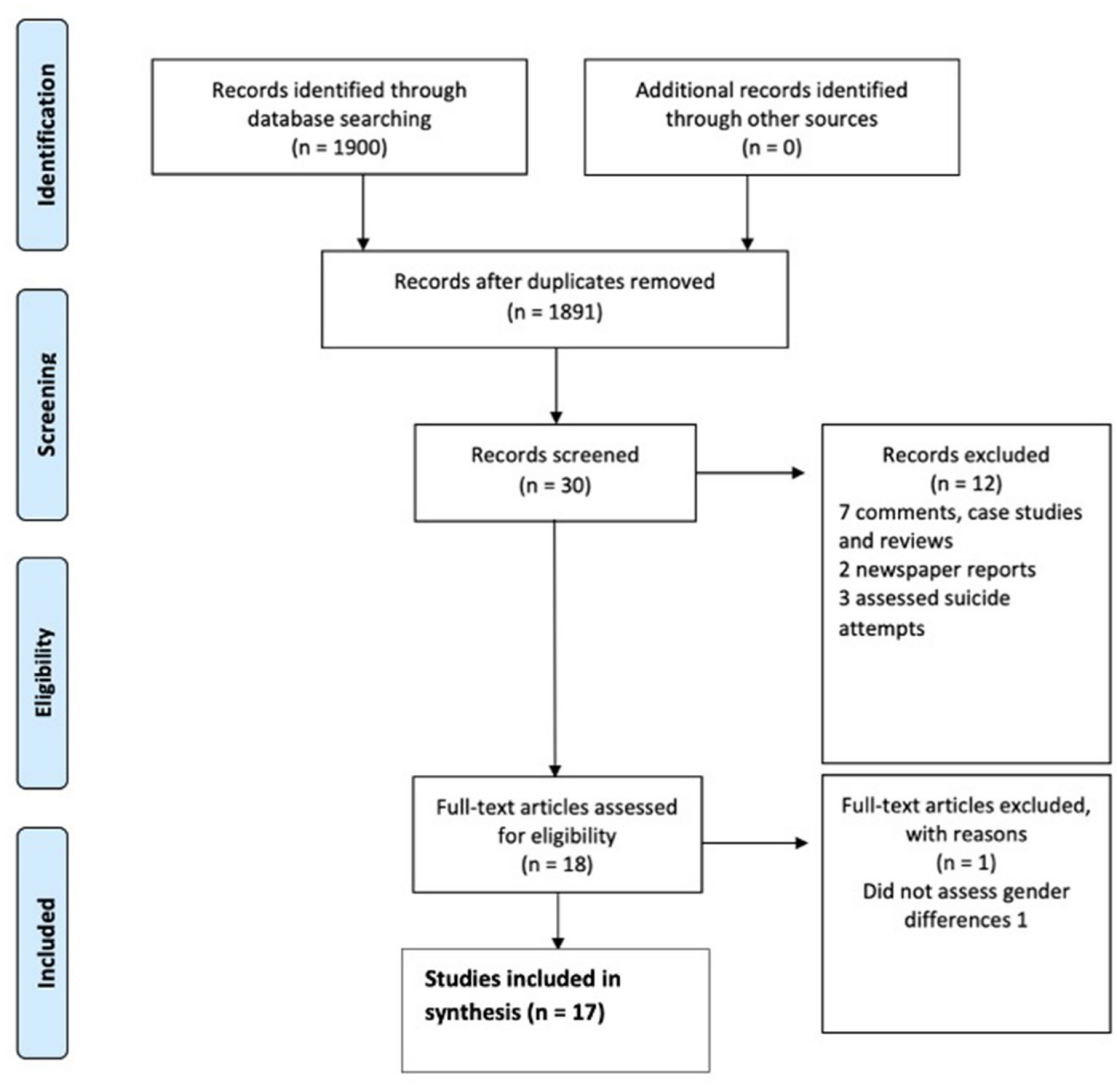
PRISMA flow diagram of study selection.

The PRISMA study selection flowchart is provided in Figure 1. A total of 1900 studies were retrieved from database searching, with 1474 studies from Embase and 426 studies from PsycINFO.

### Study Characteristics

A total of 17 studies were included in this review. Supplementary Table 1 shows the characteristics of the studies selected. Most studies originated from South India (two from Andhra Pradesh and one from Tamil Nadu), with two studies from North, East, and Central India and one from the North-East (Sikkim). The studies predominantly made use of a cross-sectional study design (82% of studies). Seven studies used data from the National Crime Records Bureau (NCRB) database, which includes data from all parts of the country. NCRB data is compiled through police records based on First Information Reports (FIRs). FIRs of unnatural deaths state the apparent cause of death based on the collection of evidence, and autopsy reports where available. Although these reports gather data from every state and originate from official reports, they cover only around 25% of deaths in rural India (17, 18). Hospital records and autopsies were the second most common sources of data, followed by police records. Of these, three studies used police records, while one study used a combination of autopsy records and community surveillance. One study included data from multiple sources, as part of the Global Burden of Diseases (GBD) study (9). The sample size of included studies ranged from 14 to 2036, with a median sample size of 230.

### Rates of Suicide

Out of the seventeen studies that were included in the review, fifteen studies reported suicide rates (Supplementary Table 1). Out of these, two did not report gender differences. These studies used different methods to analyse rates of suicide, therefore making comparisons difficult. Suicide rates among men were higher than rates for women in all the thirteen studies that reported gender differences. The GBD study, which utilised multiple data sources for estimating cause-specific mortality in India, found that suicide was the leading cause of death in the age group of 15–39 years for women, and the second leading cause of death among men in the same age group (9).

### Methods of Suicide

Out of the twelve studies that reported methods of suicide, nine reported gender differences (Supplementary Table 1). Six studies reported hanging as the most common method of suicide for both males and females (19–24). In contrast, a study by Rawat et al. (25) found self-immolation to be the most common method (50.6%) among females. This study also found that poisoning was the second most common (47.1%) method among females, and the most common (82.6%) among males. Access to the means of suicide could potentially explain this difference; this study was conducted in a rural region, where women have better access to combustion fuels used in cooking, while men, a majority of whom are engaged in agricultural work, have access to pesticides and agrochemicals (25). Similarly, poisoning was found to be the most common method of suicide for both males and females in Godavari (18), a district where agricultural labour is the most common occupation.

### Sociodemographic Factors

#### Age

Both males and females within the age group of 18–35 (24) and 15–29 (9) were more likely to die by suicide compared to older (35 and above) age groups (Supplementary Table 1). Studies tend to report higher suicide rates among younger females, with ages ranging from 11–20 (20), to 18–30 (25). For males, the highest rates of suicide have ranged from 21–30 years (20) to 35–45 years (25).

#### Marital Status

Suicide rates were higher among males and females who are married (Supplementary Table 1). This ranged from 31% (26) to 66% (21) of all suicide decedents in the study. Among the married decedents of suicide, the male:female ratio was 1.71:1, while for the unmarried, it was 3:1 (27). Only one study reported that suicide was slightly higher among married males compared to unmarried females (28).

#### Education and Employment

This review found that females who die by suicide are more likely to be literate (28) and have a secondary education (19) (Supplementary Table 1). They are also likely to be a housewife (19, 24, 29), or unemployed (28). Dandona et al. found that a majority (57.4% on average) of female deaths by suicides were among housewives, and this rate remained stable for a decade (2001–2010) (29). Males who die by suicide were more likely to report having a secondary education (19) and be employed as a daily wage-worker (24), farmer (24, 29), or be self-employed (24, 29). The suicide rate was higher in those with a secondary education for both males (27.99%) and females (28.68%), compared to graduates and those with primary education (19). Contrary to what was seen in females, suicide was more commonly reported among employed males (28).

### Suicide in Specific Populations

There were two studies that looked at suicide in specific populations—Bardale and Dixit (30) examined suicide among prisoners, and Mohandoss and Thavarajah (31) studied suicide among Indians with cancer. Bardale and Dixit (30) found that over the years 2009 to 2011, approximately 27.48% custodial deaths were recorded as cases of suicide, which is higher than the suicide rate seen in the general population. A majority (92.85%) of these deaths were male. This can be explained by the fact that only a small percentage (3.6%) of the Indian prison population is female (32). Similarly, the rate of suicide among Indians living with cancer were found to be 1.4 times higher compared to the general population and males living with cancer had a higher suicide rate compared to females, although this rate is steadily decreasing (31). This decrease in suicide rates has been attributed to improving quality of treatment and prognosis for cancer compared to earlier decades.

### Antecedent Factors

Seven studies assessed reported antecedent factors (Supplementary Table 1). Of these, only four assessed gender differences. Among males, family problems, drug abuse or addiction, physical and mental illness (19) were most commonly reported as antecedent factors. For females, marital disharmony (21), family problems, mental illness, and failure in exams have been reported as antecedent factors (19).

### COVID-19

One study (24) examined the effect of Covid-19 on suicide rates in India (Supplementary Table 1). A total of 59 deaths by suicide were observed in the first study between 21st March and 30th May 2020, during the first period of lockdown. Hanging was the most common method for males and females during lockdown. Those between the ages of 18 to 35 were most likely to die by suicide during this time. Financial loss, unemployment, poverty and hunger were identified as antecedent factors for suicide in males, while anxiety and depression, domestic conflict and violence were reported as antecedent factors for females. Comparisons to suicide rates before the pandemic could not be made, as this study did not report suicide rates observed in this region prior to the lockdown.

## Discussion

### Key Findings

The results of this review provide an overview of the literature on gender differences in suicide in India. This review also aimed to identify gaps in the literature. It was found that the suicide rate for males was higher than the rate for females in all studies that reported gender differences. This review identified hanging as the most common method of suicide for both males and females. Suicide rates were particularly high among women in the younger age group (15 to 39 years), men and women with a secondary education, married individuals, housewives, and employed men.

### Rates of Suicide

Given methodological differences in how the rates of suicide were calculated, it was difficult to make comparisons between studies in this review. However, most studies reported a higher proportion of male suicide decedents. In 2016, the suicide rate in India was estimated to be 16.5/100,000, which was higher than the global average of 10.5/100,000 (1). India accounts for 36.6% of global suicide deaths in women and 24.3% among men, despite accounting for only 17.8% of the global population (9). The suicide ratio for women in India (14.7 per 100,000) is 2.1 times higher than the global average, while for men the suicide ratio (21.2 per 100,000) is 1.4 times higher than the global average (9).

The suicide rate in older adults (95 years and older) were high for both men and women. Suicide rates in older men were higher than those seen in older women—one study reported that the suicide rate was 80.8 per 100,000 in men, and 40.6 per 100,000 in women (9). This is consistent with the data from around the world which reports high suicide rates among older adults (2), although this study also reported that a large proportion (around 71%) of suicide in India was in the age group of 15–39 years. The suicide rate was higher in women compared to men of the same age for younger age groups (14 and below to 29 years) for the majority of studies that reported age-disaggregated data. The percentage of total deaths due to suicide is also higher in women than men among young adults (15 to 29 years) (9). This is different from suicide rates seen in Western HICs, where men generally have a higher suicide rate (19.9 per 100,000) compared to women (5.7 per 100,000) (2), thus highlighting the importance of identifying age-disaggregated gender differences in the suicide rate in countries of differing income levels.

### Methods of Suicide

Similar differences were found for methods of suicide in Indians. Firearms and hanging account for a higher proportion of suicide deaths across the globe (33, 34), since these methods have higher lethality (35). An earlier systematic review on suicide in India reported that men use more violent methods of suicide, such as jumping in front of trains and firearms (11). This is consistent with data from the WHO mortality database, which showed that hanging and firearms are commonly used methods of suicide among men (36). Globally, women are more likely to die from poisoning (37). However, a majority of studies in this review found that hanging was the most common method of suicide among men and women in India, followed by poisoning. These finding challenge assumptions of gender differences in the choice of method of suicide in HICs. For example, a common assumption is that women use poisoning, a less lethal method, as their intent to die is low and it increases the possibility of intervention (38). This is challenged by the finding in this review that hanging, a method that is considered more “masculine” and lethal, is common among both males and females. Another explanation for the method of suicide that has been proposed in HICs is the concern regarding bodily disfigurement, and female suicide attempters' desire to avoid this either due to social expectations for female physical appearance, or because of concern that loved ones might find their mutilated figure (38). This assumption is also challenged in this review, since hanging and self-immolation were reported in female suicides, with the latter having a high proportion of female decedents.

Another assumption in HICs is that gender norms decrease the likelihood that women will have access to, or be familiar with methods of suicide with a higher lethality such as firearms (38). The state-specific differences in suicide methods found in this review indicates that this assumption might be relevant in the Indian context. Regional differences, such as the proportion of the population engaged in agricultural work and state-level laws that restrict the use of pesticides could explain this pattern. Poisoning is the leading method of suicide in predominantly agricultural states due to accessibility of pesticides used in farming, whereas hanging was the most common method of suicide in less agricultural states (29). One study found that restriction of access to pesticides was accompanied by a decline in suicide by poisoning, and an increase in suicide by hanging (23, 39). Hospital- or community-based studies can provide more relevant, localised information about methods of suicide and its relation to sociodemographic characteristics such as occupation.

### Sociodemographic Factors Associated With Suicide

This review found that suicide rates were higher among married people, both men and women. This is in contrast to the review by Rane and Nadkarni (11), which found that suicide was higher among married women. This could indicate a change in the demographic pattern for studies published after 2014. Nevertheless, both results contradict risk factors seen in HICs such as the United States, where the risk of suicide is lower in married individuals (40). The most common antecedent factors for suicide identified in this review are marital and family problems. This, supports Rane and Nadkarni's findings, thus highlighting the importance of cultural issues surrounding marriage and family, and its potential influence on suicide in India. However, it is unclear whether “family problems” and “marital problems” are perceived distinctly for different genders. A study of suicide in LMICs and HICs found that women had relatively higher suicide rates in countries where laws were discriminatory against women—for example, countries where women had unequal rights in family law with regard to divorce and inheritance, limited access to land and non-land assets, etc. (41). It is possible that these legal issues could also cause or be the result of “family problems” for women. Previous studies have also suggested that the higher suicide rate in younger women could be a result of domestic violence and demands for dowry from the husband's family (42). Suicide is more common 1 to 5 years after marriage, implying that women go through a period of tolerance before “seeking escape” through suicide (42). Alcoholism is another factor that could impact men and women differently—among men, alcoholism led to conflicts with family and financial issues, while among women, alcoholism was reported in the context of their husband's drinking and subsequent exposure to domestic violence (43). This suggests that the term “marital problems” and “family problems” could have different meanings for men and women. However, due to the lack of qualitative studies, there were no studies in this review that discussed how family problems could be distinct for men and women, and whether discriminatory laws influenced suicide rates in women.

Suicide rates were higher among men who were self-employed or employed as a daily wage worker. For women, suicide rates were higher among housewives. This may reflect demographic factors rather than risk factors, as men are more likely to report being self-employed or engaged in daily wage work, while women are more likely to be unemployed or housewives (44).

However, the data on the relationship between education and suicide is more conflicting. While previous studies have found that for both men and women in India, illiteracy and lower levels of formal education were associated with a high risk of suicide (45–47), this review found that suicide was higher among those with a secondary education compared to those who had only completed primary education or had completed higher secondary schooling. This supports findings by Arya et al. (48), who identified a trend of lower suicide rates among states with lower levels of literacy, and a nationally representative survey by Maselko and Patel (45) which also identified higher suicide rates in those who completed higher education, compared to those who had completed primary-level schooling (49). This could potentially be explained by geographic differences, as educational attainment varies widely within different states and districts in India (50).

### Gaps and Limitations in the Literature

This review found several limitations in suicide literature in India. First, comparisons between studies were difficult because of the differences in methodology and analysis. For example, some suicide rates were age-adjusted, while most were not, and several studies did not report gender differences for rates, methods, risk factors and antecedent factors. Only variables that were disaggregated by gender from these studies could be included. Additionally, the NCRB database is the most commonly used reference for suicide rates in India, and a majority of the studies identified in this review utilised figures from this database. There are several limitations to the data available on rates and methods for suicide through this source. The NCRB data is not collected for the primary purpose of suicide surveillance, and is dependent on information collected through FIRs submitted to the police. This information is often unreliable, since deaths by suicide are often reported as illness or an accident to avoid registering a case with the police (51). Families may also forgo reporting to avoid post-mortem examinations, due to the fear of mutilation of the body and stigma associated with suicide (51). Additionally, a scoping review on suicide in South Asia (7) found that a nationally representative cause of death survey reported significantly higher suicide rates compared to the figures published in the NCRB. This survey found that the NCRB underestimates suicide deaths in men by at least 25% and women by at least 36% (49). This was also reflected in the GBD study, which utilises data based on verbal autopsies and community surveillance programmes (52). This study found that the NCRB generally reported lower suicide rates compared to the GBD study, especially for females and in the youngest (15–29 years) and older age groups (≥60 years). Older adults in India predominantly live in rural areas, where under-reporting of suicide is common. Arya et al. (23) also propose that under-reporting of suicide in this population may occur due to the misclassification of suicide as accidental death due to overdose on prescription medication (52). Therefore, it is possible that the higher suicide rates observed in this review for males and younger individuals can be attributed to the method of data collection. The suicide rates for females may exceed those found in their male counterparts for different age groups when data is extrapolated from multiple relevant sources, as confirmed by the GBD study and the survey conducted by Patel and colleagues.

Contradictory results were also found for methods of suicide. Some studies in this review have concluded that poisoning is the most common method of suicide, based on NCRB data. However, studies in this review that report suicide data from police records and hospital records identified hanging as the most common method of suicide for men and women. This, supports findings from a systematic review on methods of suicide in South Asia (53), which found that hanging was the most common method in India over two decades (2001–2020). Therefore, studies that examine gender differences using suicide data from the NCRB should be interpreted with caution.

Second, there have been few studies that examine the prevalence of suicide among the transgender community in India. It is estimated that around 50% in this group have attempted suicide before the age of 20 (54). The lack of literature on suicide among transgender individuals is especially concerning given the number of risk factors that this group is exposed to; previous studies have documented experiences of discrimination and violence that transgender persons face in multiple social and institutional contexts (55). Although the NCRB uses categories of male, female, and transgender for suicide data, most studies in India treat gender as binary. Moreover, the reporting of suicide data is dependent on reports from family members, and these reports may not contain accurate data about a person's sexual or gender identity. Since there are several terms to describe different gender identities in India (56), research needs to be more inclusive to develop a better understanding of suicide among those who identify outside the gender binary.

Third, there were few studies that looked at various risk and protective factors. Although most of the studies in this review identified demographic factors related to suicide, there were few studies that specifically examined the role of potentially important risk factors that have been identified in studies from HICs. For example, research on protective factors from HICs (6) and LMICs (57) indicate that suicide rates are lower during pregnancy and the postpartum period. Data from the GBD study indicates that deaths attributable to suicide in pregnant women from the Southeast Asia region were lower than suicides among all deaths of women in this region (57). Moreover, gender differences in potential risk factors such as interpersonal violence, caste-based discrimination, and psychiatric diagnoses were not reported in the studies identified in this review. This could be explained by the fact that several studies used data from the NCRB, which does not include information on psychiatric illnesses and mental health issues, previous suicide attempts, or substance use. Other studies from India that use psychological autopsy method have associated suicide with psychosocial stressors, psychiatric illnesses, and alcohol use disorders (58). Further studies in the Indian context, particularly those that collect information through hospital records and psychological autopsies, or utilise a case-control design, are necessary to understand whether factors such as motherhood and pregnancy, psychiatric diagnoses etc can act as risk or protective factors in the Indian population.

Scoping reviews are especially suitable for addressing region-specific issues where availability of data may be limited. This review had the strength of providing a comprehensive overview of recent literature on gender differences in suicide in India. This review provided recent evidence that supports the findings of Jordans, et al. (7) and Rane and Nadkarni (11), both of which identified high rates of suicide among young people, especially women. Additionally, this review identified several gaps in the literature. Some limitations of this scoping review are important to note. Due to the variation in reporting of suicide data, a meta-analysis could not be conducted, although this is not recommended for scoping reviews (13). Moreover, restricting the review to a specific time period meant that changes in suicide data over decades could not be assessed. However, this enabled the review to uncover recent data on gender differences in suicide. For example, the GBD study identified that although female suicide rates have declined since the 2000s, the proportion of global suicide deaths in India has increased more for women than for men. Additionally, the timeline and resources for this review allowed for the search of only two electronic databases. It is possible that some relevant studies, including qualitative studies on gender and suicide, could have been identified if more databases had been included in the search process.

### Recommendations

These findings have important implications for future research. For instance, suicide surveillance systems need to be established in India in regions where they currently do not exist. Nationally representative surveys of suicide, such as the one conducted as part of the GBD study (29), are important in understanding suicide at the national level. Additionally, the reported suicide rates in India were found to be high compared to the global average, especially for younger women. Further research is necessary to address the gaps in the current literature, including gender-specific risk and protective factors for suicide.

### Conclusion

Suicide is particularly concerning in LMICs such as India, where resources to handle suicide prevention are scarce. A scoping review of research on suicide and gender in India was conducted. This review focused on rates of suicide, methods of suicide, risk factors and antecedent factors for suicide. The suicide rate and proportion of deaths by suicide were particularly high in young women. Hanging was found to be the most common method of suicide. This review also identified several gaps in literature, such as the lack of literature on individuals who identify outside the gender binary of male and female, and the need to understand gender differences in risk and protective factors for suicide.

## Author Contributions

PR wrote the manuscript. PT, RM, RR, and CR contributed to manuscript revision and editing. All authors contributed to the conception and planning of the scoping review.

## Funding

PR was funded by the Global Challenges Research Fund. The funders had no role in study design, data collection and analysis, decision to publish, or preparation of the manuscript.

## Conflict of Interest

The authors declare that the research was conducted in the absence of any commercial or financial relationships that could be construed as a potential conflict of interest.

## Publisher's Note

All claims expressed in this article are solely those of the authors and do not necessarily represent those of their affiliated organizations, or those of the publisher, the editors and the reviewers. Any product that may be evaluated in this article, or claim that may be made by its manufacturer, is not guaranteed or endorsed by the publisher.
