# Supplementary material for: A Scoping Review of Gender Differences in Suicide in India
Source: Front Psychiatry. 2022 May 20;13:884657. doi: 10.3389/fpsyt.2022.884657 (PMC9165759; doi:10.3389/fpsyt.2022.884657)
Supplement: Supplementary file 1 [file Table_1.docx]

Supplementary Table 1. Characteristics and findings of included studies

| **Study and Year** | | **Objective** | **Source of Data** | **Study Design** | **N** | **Rates of Suicide** | **Method of Suicide** | **Risk factors and**  **sociodemographic factors** | **Antecedent factors** |
| --- | --- | --- | --- | --- | --- | --- | --- | --- | --- |
| **Armstrong et al.** | 2019 | To systematically investigate whether mass media reports of suicides reflect the epidemiological data on suicide in a high suicide state in India. | NCRB  (June - December, 2016) | Cross-sectional analysis and content analysis | NA | Males = 68%  Females = 32% | Hanging = 6471 (of  which 70% male);  Poisoning  = 6035 (of  which 70% male)  Self-immol ation = 1610 (of  which 40% male) | Most common age groups for suicide completers:  Females: 18-29 years; Males:  30-44 years  Most common marital status for suicide completers:  Married for both sexes (50% of male decedents, 23% of female decedents); | Gender differences not reported. |
|  | |  |  |  |  |  |  |  |  |

| **Study and Year** | | **Objective** | **Source of Data** | **Study Design** | **N** | **Rates of Suicide** | **Method of Suicide** | **Risk factors and**  **sociodemographic factors** | **Antecedent factors** |
| --- | --- | --- | --- | --- | --- | --- | --- | --- | --- |
| **Arya et al.** | 2019 | 1. Identify national and regional patterns in method-specific suicide rates; and 2. Identify social, demographic and economic correlates of state-level variation in suicide methods. | NCRB (2001 -  2014) | Cross-sectional | NA | Method-specific suicide rates assessed. | Hanging = Males (35.07%);  Females (29.42%)  Insecticide  = Males (18.31%);  Females (17.37%)  Self-immol ation = Males (4.57%);  Females (15.74%) | 2x to 4x higher suicide rates in more economically developed states for both sexes.  Agricultural states had higher rates of insecticide poisoning for both sexes.  Higher rates for hanging among males in less agricultural states.  Higher rates of suicide by  self-immolation among females in more agricultural states. | Gender differences not reported. |

| **Study and Year** | | **Objective** | **Source of Data** | **Study Design** | **N** | **Rates of Suicide** | **Method of Suicide** | **Risk factors and**  **sociodemographic factors** | **Antecedent factors** |
| --- | --- | --- | --- | --- | --- | --- | --- | --- | --- |
| **Arya et al.** | 2021 | To determine whether decreases in suicide by insecticide poisoning were offset by increases in suicide by other methods. | NCRB (2001-2014  ) | Longitudinal | NA | Method-specific c suicide rates assessed. | Males = 63% of fall in suicide by insecticide poisoning and other methods were offset by hanging; 29% were offset by rises in other poisoning. Females = 25% of the decrease in suicides by insecticides and all other methods were offset by rises in suicide by hanging, and 35% were offset by rises in other poisoning | Gender differences not reported. | Gender differences not reported. |

| **Study and Year** | | **Objective** | **Source of Data** | **Study Design** | **N** | **Rates of Suicide** | **Method of Suicide** | **Risk factors and**  **sociodemographic factors** | **Antecedent factors** |
| --- | --- | --- | --- | --- | --- | --- | --- | --- | --- |
| **Badiye, et al.** | 2014 | The objective of the current study was to analyze the trends of suicide mortality in the Nagpur city of Maharashtra State in India. | Police records from Nagpur, Maharashtr a (January 2009 to December 2013) | Cross-sectional | 2036 | Males = 71.42%;  Females = 28.58% | Hanging = 1216 (of  which 74% male);  Insecticide poisoning = 371 (of  which 73% male)  Drowning= 329 (of  which 82% male)  Self-immol ation = 284 (of which 34% male) | Most common age groups for suicide completers:  Females:15-29 years;Males:30-44 years  Most common marital status for suicide completers: Marrie d for both sexes (males 66.73%,  females 56.45%);  Most common occupations for suicide completers:Males: Private sector (61.14%);  Females: Housewife (65.86%) | Males = Family problems (38.25%), drug abuse/addiction (19.98%), other prolonged illness (11.96%),  mental illness (6.68%);  Females = Family problems (52.65%),  mental illness (9.10%), other prolonged illness and failure in exams (both 8.95%) |

| **Study and Year** | | **Objective** | **Source of Data** | **Study Design** | **N** | **Rates of Suicide** | **Method of Suicide** | **Risk factors and**  **sociodemographic factors** | **Antecedent factors** |
| --- | --- | --- | --- | --- | --- | --- | --- | --- | --- |
| **Bardale and Dixit** | 2015 | The present study was undertaken to assess the trends of suicide in custody and to identify characteristics that can be utilized to prevent such deaths. | Hospital records from Nagpur, Maharashtr a (2001 to  2010) | Cross-sectional | 14 | Males = 93%;  Females = 7% | Most common methods for suicide completers:  Females = poisoning  Males = hanging, poisoning | Gender differences not reported. | Gender differences not reported. |

| **Study and Year** | | **Objective** | **Source of Data** | **Study Design** | **N** | **Rates of Suicide** | **Method of Suicide** | **Risk factors and**  **sociodemographic factors** | **Antecedent factors** |
| --- | --- | --- | --- | --- | --- | --- | --- | --- | --- |
| **Chettri, et al.** | 2016 | To statistically analyze suicidal data from Sikkim with the focus on throwing light on the vulnerable groups. | Police records from Gangtok, Sikkim (2006-2015  ) | Cross-sectional | 1604 | Males = 65.5%  Females = 34.5% | 2015:  Hanging = 157 (of  which 66% male)  Jumping = 9 (of which  89% male)  Stabbing = 1 (of which  100%  male)  Drowning  = 1 (of which 100%  male) | 2015:  Most common age groups for suicide completers:  Males: 21–30  Females: 11–20 | Gender differences not reported. |
|  | |  |  |  |  |  |  |  |  |

| **Study and Year** | | **Objective** | **Source of Data** | **Study Design** | **N** | **Rates of Suicide** | **Method of Suicide** | **Risk factors and**  **sociodemographic factors** | **Antecedent factors** |
| --- | --- | --- | --- | --- | --- | --- | --- | --- | --- |
| **Choudhury** | 2020 | To analyse the role and magnitude of severity of factors contributing to suicides. | Observatio nal analysis from Lucknow, Uttar Pradesh (24th March - 31st May 2020) | Observational analysis | 59 | Males = 55.9%  Females = 44.1% | Hanging = 55 (of  which 58% male);  Drowning  = 2 (of  which 50% male);  Poisoning  = 2 (of which 100%  female) | Highest suicide rate in age 18-35 years for both sexes.  Most commonly reported occupation of deceased:  Females: Housewives, domestic help, students;  Males: Daily wage worker, businessperson/self  -employed, farmer | Females - anxiety and depression, domestic conflict, violence  Males - Financial loss or unemployment, poverty, hunger |
|  | |  |  |  |  |  |  |  |  |

| **Study and Year** | | **Objective** | **Source of Data** | **Study Design** | **N** | **Rates of Suicide** | **Method of Suicide** | **Risk factors and**  **sociodemographic factors** | **Antecedent factors** |
| --- | --- | --- | --- | --- | --- | --- | --- | --- | --- |
| **Dandona, et al.** | 2017 | (i)to highlight variability in the reported magnitude of suicide over time and within country, using the NCRB data. (ii) to draw attention to the reasons for and means of suicide, and to highlight the variability within countries and by demographic groups that could facilitate identification and prioritization of immediate actions for suicide prevention. (iii) to discuss the limitations of the available NCRB data for surveillance, to address the gaps in data to improve surveillance data quality. | NCRB (2001-2010  ) | Longitudinal | NA | Total = 14.9 to  15.4 suicides per 100000 population (estimated increase of 0.0405 suicides/year);  Males = 17.8 to  19.5 (estimated increase of 0.138/year);  Females = 11.9  to 11.1 (estimated decrease of 0.062/year) | Females: self-immolation was the third-ranking means of suicide (16.3% on average) | Most commonly reported occupation of deceased:  Females: Housewives (57.4%)  Males:  Self-employed (20%), agricultural  (20%) | Gender differences not reported. |
|  | |  |  |  |  |  |  |  |  |

| **Study and Year** | | **Objective** | **Source of Data** | **Study Design** | **N** | **Rates of Suicide** | **Method of Suicide** | **Risk factors and**  **sociodemographic factors** | **Antecedent factors** |
| --- | --- | --- | --- | --- | --- | --- | --- | --- | --- |
| **Dandona, et al.** | 2018 | To identify time trends of suicide deaths, and the heterogeneity in its distribution between the states of India from 1990 to 2016. | GBD Study (1990 to  2016) | Cross-sectional | 230314 | Total Suicide Death Ratio (SDR) = 17·9 (95% UI  15·0–19·4) per  100000  population;  Males = 135934 (95% UI 94305–151239  );  Females = 94380 (95% UI  84002–104274  ) | Gender differences not reported. | Suicide rate highest among 15-29 years for both sexes. | Gender differences not reported. |
|  | |  |  |  |  |  |  |  |  |

| **Study and Year** | | **Objective** | **Source of Data** | **Study Design** | **N** | **Rates of Suicide** | **Method of Suicide** | **Risk factors and**  **sociodemographic factors** | **Antecedent factors** |
| --- | --- | --- | --- | --- | --- | --- | --- | --- | --- |
| **Joshi et al.** | 2015 | The aim of this study was to determine the proportion of deaths attributable to suicides in 45 villages of Andhra Pradesh, India, over a 4-year period using a verbal autopsy method. | Autopsy records from Godavari, Andhra Pradesh (2003 -  2007) | Cross-sectional | 280 | Females = 28.8/100000  per year  Males = 46.4/100000  per year | Method unknown for most (41%)  suicides.  Poisoning  = 111 (of  which 70% male);  Hanging = 33 (of  which 67% male);  Burning = 12 (of  which 33%  male) | Most common age groups for suicide completers:  Males: 30–44  Females: 10–29 | Gender differences not reported. |
|  | |  |  |  |  |  |  |  |  |

| **Study and Year** | | **Objective** | **Source of Data** | **Study Design** | **N** | **Rates of Suicide** | **Method of Suicide** | **Risk factors and**  **sociodemographic factors** | **Antecedent factors** |
| --- | --- | --- | --- | --- | --- | --- | --- | --- | --- |
| **Kishore, et al.** | 2017 | To identify demographic profile and attributing factors for suicide hanging. | Autopsy records from Ranchi, Jharkhand (April 2012  - October 2013) | Cross-sectional | 351 | Males = 66.67%  Females = 33.33% | Only cases of hanging were reported. | Suicide rate highest among 15-29 years for both sexes.  53% of males and 63% of females who died by suicide were married. | Gender differences not reported. |
| **Mohandoss and**  **Thavarajah al.** | 2016 | The aim of this study was to present the brief epidemiology of completed suicides in relation to PLWC in India during the period of 2001–2014. In addition, an attempt is made to see this under the demographics feature of age group, gender, and overall illness-related suicide rates. | NCRB (2001 -  2014) | Cross-sectional | NA | 10421 (0.61%)  of all suicides were found to be  cancer-related, of which 68.7% were males | Gender differences not reported. | Suicide rate highest among 45-59 years for both sexes. | 10421 (0.61%)  of all suicides were found to be cancer-related, of which 68.7% were males |
|  | |  |  |  |  |  |  |  |  |

| **Study and Year** | | **Objective** | **Source of Data** | **Study Design** | **N** | **Rates of Suicide** | **Method of Suicide** | **Risk factors and**  **sociodemographic factors** | **Antecedent factors** |
| --- | --- | --- | --- | --- | --- | --- | --- | --- | --- |
| **Pasi et al.** | 2015 | To evaluate psychiatric traits and genetic predisposition among the family members of suicide completers. | Family members of deceased from New Delhi (2008  - 2011) | Cross-sectional | 25 | Males = 80%  Females = 20% | Hanging = Males (20%); Females(20%)  Poisoning=Males(65%); Females(40%)  Cutting wrist =Males(15%); Females(40%) | Most common age groups for suicide completers:  Males: 25–45  Females: 15–25  Higher suicide rate among married males and unmarried females; employed males and unemployed females.  Higher suicide rates among literate and those belonging to nuclear families for females | Gender differences not reported. |
|  | |  |  |  |  |  |  |  |  |

| **Study and Year** | | **Objective** | **Source of Data** | **Study Design** | **N** | **Rates of Suicide** | **Method of Suicide** | **Risk factors and**  **sociodemographic factors** | **Antecedent factors** |
| --- | --- | --- | --- | --- | --- | --- | --- | --- | --- |
| **Pradhan et al.** | 2018 | 1. To study the patterns of suicidal female deaths; 2. To analyse and identify the preventable risk factors in such deaths. | Hospital records from Chennai, Tamil Nadu (May 2013  - August 2014) | Cross-sectional | 44 | 100% female | Hanging = 24 (54 %)  Poisoning = 11 (23%)  Burns = 7 (15%)  Jumping from heights =1 (4 %)  Suicide on road=1 (4 %) | Illiterate and higher secondary-level education = 13 suicide cases (31%)  Post high-school diploma = 7 suicide cases (15%)  Primary school = 2 suicide cases (4%)  Middle class = 19 suicide cases (43%)  Lower middle class = 13 suicide cases (31%)  Upper class = 2 suicide cases | Marital disharmony = 54% of suicide cases  Infertility = 15% of suicide cases  Education = 11% of suicide cases  Financial dispute = 8% of suicide cases  Old-age loneliness = 8% of suicide cases  Inability to find a partner = 4% of suicide cases |
|  | |  |  |  |  |  |  |  |  |

| **Study and Year** | | **Objective** | **Source of Data** | **Study Design** | **N** | **Rates of Suicide** | **Method of Suicide** | **Risk factors and**  **sociodemographic factors** | **Antecedent factors** |
| --- | --- | --- | --- | --- | --- | --- | --- | --- | --- |
| **Rawat et al.** | 2018 | To characterize Suicide Deaths from the period of 2010–2015 by analyzing variables; Age, gender, suicide methods and seasonal variation for the suicide cases reported at a Tertiary care center located in Warangal district, Telangana, India. | Hospital records from Warangal, Telangana (2010 to  2015) | Cross-sectional | 1325 | Males = 56.6%;  Females = 43.4% | Self-immol ation = 415 (of which 30% males)  Hanging = 20 (of  which 35% males)  Poisoning  = 890 (of  which 70% males) | Most common age group for suicide completers:  Males: 35-45 years  Females: 18-35 years | Gender differences not reported. |
| **Singh et al.** | 2021 | To test the hypothesis that conflict between rapid economic growth and inadequate female autonomy varies positively with female suicides. | NCRB (2001 to  2011) | Cross-sectional | NA | 9.4 per 100,000  (female SDR only) | Gender differences not reported. | Greater prevalence of female suicides observed in states with greater economic development. | Gender differences not reported. |
|  | |  |  |  |  |  |  |  |  |

| **Study and Year** | | **Objective** | **Source of Data** | **Study Design** | **N** | **Rates of Suicide** | **Method of Suicide** | **Risk factors and**  **sociodemographic factors** | **Antecedent factors** |
| --- | --- | --- | --- | --- | --- | --- | --- | --- | --- |
| **Singh, et al.** | 2017 | The aim of this study was to illustrate the epidemiological profile of complete suicidal poisoning cases autopsied at Department of Forensic Medicine and Toxicology, Rajendra Institute of Medical Sciences, Ranchi, India. | Autopsy records from Ranchi, Jharkhand (April 2013  - October 2014) | Cross-sectional | 180 | Males = 55%  Females = 45% | Only cases of poisoning included. | Suicide rates higher among 15 - 29 years for both sexes;  31.7% of males  and 28.3% of females who died by suicide were married. | Gender differences not reported. |

Abbreviations: GBD = Global Burden of Diseases; NCRB = National Crime Records Bureau; SDR = Suicide Death Rate
